# P-1192. Association between adeno-associated virus 2 and pediatric acute hepatitis of unknown etiology in Japanese children

**DOI:** 10.1093/ofid/ofae631.1376

**Published:** 2025-01-29

**Authors:** Jun-ichi Kawada, Ken-ichi Iwada, Yuka Torii, Aiko Sakai, Yuto Fukuda, Kazunori Haruta, Makoto Yamaguchi, Takako Suzuki, Yuri Etani, Yoshiyuki Takahashi, Shuichiro Umetsu, Ayano Inui, Ryo Sumazaki

**Affiliations:** Fujita Health University School of Medicine, Toyoake, Aichi, Japan; Nagoya University Graduate School of Medicine, Nagoya, Aichi, Japan; Nagoya University Graduate School of Medicine, Nagoya, Aichi, Japan; National Center for Global Health and Medicine, Tokyo, Tokyo, Japan; Nagoya University Graduate School of Medicine, Nagoya, Aichi, Japan; Nagoya University Graduate School of Medicine, Nagoya, Aichi, Japan; Nagoya University Graduate School of Medicine, Nagoya, Aichi, Japan; Nagoya University Graduate School of Medicine, Nagoya, Aichi, Japan; Osaka Women’s and Children’s Hospital, Izumi, Osaka, Japan; Department of Pediatrics, Nagoya University Graduate School of Medicine, Nagoya, Aichi, Japan; Saiseikai Yokohamashi Tobu Hospital, Yokohama, Kanagawa, Japan; Saiseikai Yokohamashi Tobu Hospital, Yokohama, Kanagawa, Japan; Ibaraki Children's Hospital, Mito, Ibaraki, Japan

## Abstract

**Background:**

Outbreaks of acute hepatitis of unknown etiology (AHUE) in children were reported in Western countries in 2022. Previous studies found that the adeno-associated virus 2 (AAV2) and other viruses, such as human adenovirus (HAdV) and human herpesvirus-6 (HHV-6), were frequently detected in patients with AHUE. As HAdV and HHV-6 are known as “helper viruses” that facilitate AAV2 replication, co-infection with AAV2 and helper viruses has been postulated as a potential trigger of AHUE. However, whether hepatitis associated with AAV2 existed prior to the AHUE outbreaks in 2022 has not yet been investigated. We aimed to investigate the association between AAV2 and pediatric acute hepatitis in Japanese children as well as the incidence of AAV2-related hepatitis before 2022.

Visual abstract

**Figure:**
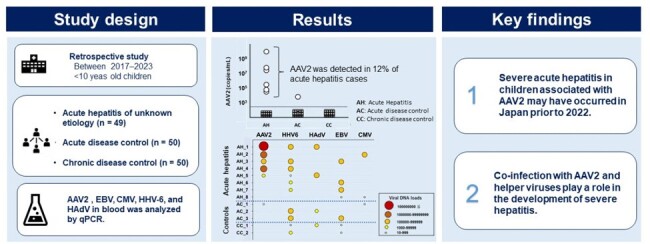

**Methods:**

Preserved blood samples from 49 pediatric patients (median age: 3 years) with acute hepatitis of unknown etiology between 2017 and 2023 were retrospectively analyzed. Blood samples from 50 children with acute illnesses, such as infectious diseases and 50 children with chronic conditions were used as controls. Viral DNA loads were quantified using real-time PCR.

**Results:**

AAV2 DNA was detected in 12% (6/49) of acute hepatitis cases (median age: 2.5 years), but in only one acute illness case and none of the chronic condition control cases. The concentration of AAV2 DNA (range: 2.5×10^3^ to 5.0×10^8^ copies/mL) in the six patients in the acute hepatitis group was higher than that in the patient in the acute-illness control case (7.8×10^2^ copies/mL). Co-infection with one or more helper viruses, including HAdV, HHV-6, cytomegalovirus, and Epstein–Barr virus, was observed in five of six AAV2-positive acute hepatitis cases.

**Conclusion:**

Our results indicate the sporadic occurrence of severe pediatric hepatitis associated with AAV2 infection in Japan prior to AHUE outbreaks in Western countries in 2022. Conversely, the detection rate of AAV2 in this study was much lower than that of AHUE in Western countries, suggesting that the outbreak of AHUE related with AAV2 is an exceptional phenomenon associated with mitigation measures against COVID-19. Our findings suggest that coinfection with AAV2 and helper viruses plays a role in the development of severe hepatitis.

**Disclosures:**

**All Authors**: No reported disclosures

